# Development and initial evaluation of the usefulness of a question prompt list to promote patients' level of information about work-related medical rehabilitation: a pilot study

**DOI:** 10.3389/fresc.2024.1266065

**Published:** 2024-02-15

**Authors:** Matthias Lukasczik, Hans Dieter Wolf, Heiner Vogel

**Affiliations:** Rehabilitation Sciences Section, Center of Mental Health, Würzburg University Hospital, Würzburg, Germany

**Keywords:** medical rehabilitation, question prompt list, patient information, patient knowledge, work-related rehabilitation, pilot study

## Abstract

**Introduction:**

The purpose of this study was to develop a question prompt list (QPL) to support patients undergoing work-related medical rehabilitation in obtaining relevant information and to explore how patients and physicians rate the QPL regarding its usefulness, practicability, and perceived (additional) effort.

**Methods:**

An initial item pool was assessed by rehabilitation patients (*N* = 3) in cognitive interviews, rated by physicians and other health professionals (*N* = 11), and then further modified. The final QPL version (16 items) was used by patients (*N* = 36) in medical admission interviews in an inpatient medical rehabilitation facility and then evaluated. Physicians evaluated the QPL after each interview with a study participant (*N* = 6; *k* = 39 interviews).

**Results:**

The QPL was used by 50% of patients who rated its usefulness and comprehensibility positively. Neither the need for information nor satisfaction with the information received was correlated with QPL use. The physicians' assessment showed a positive evaluation regarding the provision of information and structuring of the conversation, but also a higher perceived time expenditure.

**Discussion:**

While initial testing of the QPL in work-related medical rehabilitation as a tool to support patient-provider communication generally showed a favorable evaluation by patients using it and physicians, future research should address its validity and effectiveness.

## Introduction

There are a variety of tools and interventions to promote patient informedness or, in more general terms, patient-centered communication in health care (1). These include decision aids (2), patient education programs (3), or shared decision-making interventions (4).

Question prompt lists (QPL) are one potential tool for promoting patient information. QPL are sets or short lists of “key questions” related to a health- or disease-related topic, such as treatment options and their benefits or risks, prognosis, or other aspects related to the disease or therapy (5). Patients should use these questions when talking to their doctor or healthcare provider, so that they can obtain the information that is relevant to them. QPL have been characterized as a low-effort method for communicating information and promoting patient engagement (6).

QPL have been developed and evaluated mainly in the context of oncology (7). There is evidence that their use encourages the asking of questions (5, 7, 8) and satisfies patients' information needs or reduces information deficits (8, 9). Patients generally rate QPL as useful and helpful (10–13). Outside of oncology, QPL have been studied less frequently, e.g., in family medicine (14), preparation for surgery (15, 16), heart failure (17), and depression (18).

Their potential to provide patients with a chronic illness with relevant information on their disease and the skills necessary for successful disease self-management has hardly been studied. In particular, to the authors' knowledge, QPL have not yet been used or tested in the context of medical rehabilitation (MR)^1^. MR is a key setting for the treatment of chronic diseases, such as orthopedic, neurological, or cardiovascular disorders. Patients must have or acquire the necessary skills and knowledge to successfully manage their disease. Consequently, the effectiveness of MR programs is influenced by the extent to which rehabilitation patients feel adequately informed about their content and goals (including the improvement of self-management skills). Studies from Germany have shown that patients who are informed about MR according to their needs and involved in treatment planning are more satisfied with rehabilitative treatment (19–21) and are better able to cope with their individual problem situation (22).

In contrast, there are only few studies on the expectations and information needs of MR patients in Germany. Existing studies indicate that at least some MR patients do not feel (sufficiently) prepared for MR or that there are information deficits (23, 24). There is also a lack of comprehensible sources of information for patients about MR (25).

We approached this gap by exploring the usefulness of a QPL as an information aid for patients undergoing inpatient MR in a pilot study. We chose work-related medical rehabilitation (WMR) as the setting for our study. This type of program (with a regular duration of three to four weeks) is provided by the German statutory pension insurance for persons of working age with pronounced occupational problems such as longer periods of work disability/sick leave, conflicts at work or other issues that may jeopardize long-term work ability. WMR components include work ability assessment, work-related functional capacity training, work-related psychosocial groups, and social counselling with a particular focus on the work situation (26). As WMR programs currently lack standardized patient information materials and it is largely unknown whether WMR patients feel sufficiently informed about the occupational focus of their rehabilitation program, this seemed a suitable setting for testing a QPL in MR.

Against this background, the objective of our study was to explore the perceived usefulness and applicability of a QPL to help WMR patients obtain relevant information about the work-related focus of WMR at the start of treatment.

We addressed the following research questions:

1. How do WMR patients at the start of their rehabilitation rate the QPL after the medical admission interview in terms of its perceived usefulness, comprehensibility, and satisfaction with the information received?
2. How do physicians in WMR centers evaluate the QPL in terms of its perceived practicability, perceived (additional) effort, and perceived benefit for structuring the interview and providing important information?

## Materials and methods

### Study design

We conducted a two-phased mixed-methods pilot study. In the first phase, a draft QPL was developed, presented to WMR patients during cognitive interviews and subsequently adapted. In the second phase, the instrument was evaluated by WMR patients and physicians using questionnaires immediately after the physician admission interview, in which the patients used the QPL.

As data collection in both phases coincided with various restrictions in (rehabilitation) hospitals due to the Covid-19 pandemic, including the dropout of one out of two MR facilities from the study, a smaller number of participants than planned could be included in both study phases.

The study was approved by the ethics committee of the University of Würzburg. Written informed consent was obtained from all individual participants for being included in the study.

### Study procedures and participants

First phase: Instrument development.

Relevant literature on the topic was reviewed. Specifically, the WMR profile of requirements of the German pension insurance scheme, available German-language studies on the information needs of MR patients (including an unpublished qualitative survey of WMR patients and clinicians in two MR facilities conducted by the authors), and a patient information website on MR preparation (www.vor-der-reha.de; originally developed in a research project at the University of Lübeck, Germany; information only available in German) were consulted. From these sources, information was extracted about what WMR-related content patients should know or learn at the start of a WMR program, what additional general information on MR might be relevant, and what information needs related to MR (“regular”/standard MR as well as work-related programs) have been documented in studies.

In addition, a search was performed in the MEDLINE/PUBMED databases using the search term combinations +information +need* AND rehabil* and +question* +ask* AND rehabil* (search date: 4 February 2020). Eight potentially relevant publications were identified and screened but their content proved not to be relevant to the study.

On this basis, items (in question form) for the QPL were formulated independently by two researchers. These were then compared and merged. The resulting initial item pool comprised 59 questions on the topics “what is WMR”, “treatment components”, “occupational focus”, “treatment goals”, “treatment efficacy”, “rehabilitation patient's own role”, and “other”. After all questions had been checked for redundancy, relevance, and comprehensibility, the number of items was reduced to 23 questions.

In the next step, the questions were presented to WMR patients during cognitive interviews (27) to assess their comprehensibility. The interviews were conducted jointly by two researchers, recorded, and supplemented by a handwritten protocol (verbal protocols). Guidelines and protocol sheets for the cognitive interviews had been previously developed.

Potentially eligible patients (inclusion criterion: participation in a WMR program; exclusion criteria: age under 18 or over 65 years, severe cognitive impairment (in terms of inability to participate in the interview with regard to reading or understanding the QPL items as intended due to neurological, intellectual or other impairments), lack of understanding of the German language, post-acute follow-up treatment) were approached at the rehabilitation center and asked to participate in the study. An appointment for the interview was coordinated and appropriate rooms were made available on site. Each interview was conducted jointly by two researchers and recorded as well as documented on a protocol sheet.

23 items were discussed one by one with the participant; each item was read out loud. The participant was then asked to explain the extent to which the question was understandable to them, was perceived as important and relevant (in retrospect), and whether there were any suggestions for rephrasing the question. For individual items, the interviewers also asked specifically how the central terms or keywords were understood (“probing”). The qualitative data collected were compared with the protocol sheets and supplemented as necessary. For each QPL item, the participants' comments on “comprehensibility of the question,” “importance/relevance of the question,” and “suggestions for rephrasing” were evaluated. Where feasible, they were included in an adaptation of the wording of the item in question. If there were indications of duplication, overlaps or redundancies, the items concerned were checked to see whether they could be merged or whether a rewording or deletion was appropriate. This resulted in the deletion of four items.

Interview data were collected between July and October 2020. The target sample size for the interviews was *N* = 10–16 persons in two MR centers.

Then the staff responsible for the treatment of WMR patients from two inpatient MR centers were asked to provide an open/free-text written assessment of the fit and comprehensibility of the items. Their feedback was incorporated into the draft QPL where appropriate. The revised version was made available to them, approved by them and then finalized.

The final QPL version included 16 items. These are listed in Table 1.

**Table 1:** Items of the question prompt list.

1	What does WMR mean?
2	Why is WMR important to me in particular?
3	What therapies are provided in WMR (as opposed to standard medical rehabilitation)?
4	Who can help me in WMR when it comes to sick pay, pension, and other things?
5	Can I also recover during WMR?
6	Do I receive information and feedback from diagnostic and therapeutic measures?
7	Can I also say what my problems are at work from my point of view?
8	How well does WMR fit my work situation?
9	How can WMR help me with problems with my work situation? *For example: How do I get back to work? How can I deal with stress? How can WMR help me if I have lost my job?*
10	Do I have a say in what treatment/measures I receive in WMR?
11	What can I do myself to make sure that WMR helps me and is successful?
12	Are there other facilities involved in WMR besides the rehabilitation clinic?
13	Who else will be informed about my WMR program besides me? *For example: My employer, my family doctor, the company doctor*
14	What happens after WMR?
15	Should I continue treatment after WMR?
16	If I still have questions or problems after WMR: Who is responsible for me then?

Second phase: QPL assessment/evaluation by WMR patients and physicians.

The QPL was mailed in advance to rehabilitation patients before the start of a WMR program in an inpatient medical rehabilitation center (treatment focus: musculoskeletal disorders), along with a patient information sheet and an informed consent form. Those patients who consented to participate in the study were given the evaluation questionnaire and were asked to complete it after the medical admission interview. The QPL was used during admission because patients should be provided with information considered relevant to successful completion of the program at the beginning of a WMR program.

Participating physicians completed their questionnaire after the interview with a study participant during the same period.

Participation in a WMR program was defined as the inclusion criterion for the recruitment of study participants. Exclusion criteria were an age below 18 or above 65 years, severe cognitive impairments, a lack of understanding of the German language, and a post-acute follow-up treatment. Consecutive sampling took place between January and November 2021. On the medical side, physicians who conduct the medical admission interview and inform patients about the goals and contents of WMR were included.

The target sample size for patients was *N* = 80^2^. No sample size was specified for physicians. We sought to include all physicians who regularly conducted medical admission interviews at the MR center.

Given the pilot nature of the study, the patient questionnaire used in the formative evaluation of the QPL was developed specifically for this study. Items were formulated on the perceived QPL usefulness and comprehensibility of the QPL (item examples: “The QPL helped me think of everything important when talking to the doctor”; “The QPL questions were easy for me to understand”; scored as „fully agree“; „somewhat agree”; “somewhat disagree”; “fully disagree”). The actual use of the QPL in the medical admission interview was also assessed. In addition, information needs prior to the start of the WMR program (retrospective assessment, 4-point scale ranging from “very high” to “very low”) and the level of satisfaction with the information received (4-point scale ranging from “very satisfied” to “very unsatisfied”) were assessed.

The CARE scale for the assessment of perceived physician empathy (German version) (28) was used to assess patient-centeredness in the physician admission interview. It includes 10 items rated on a 5-point scale (ranging from “fully agree” to “fully disagree”). Socio-demographic and socio-medical variables (age, gender, educational and employment status) were compiled.

The physician questionnaire was also developed specifically for the study. Items were formulated on the perceived use of the QPL by the patient, the perceived QPL usefulness for conveying relevant information and structuring the conversation, its perceived practicability, and the perceived (additional) effort (4-point scale ranging from “fully agree” to “fully disagree”). In addition, experience with QPL or comparable instruments and basic sociodemographic information including professional experience in the field of MR and WMR (in years) were assessed. A skip rule was included in the questionnaire, depending on whether the QPL had been used by the study participant in the interview or not. In the latter case, the items relating to the (hypothetical) presumed benefit for the interview should be completed (actual QPL use, e.g., “Did the QPL contribute to…” vs. hypothetical QPL use, e.g., “Would the QPL have contributed to…”).

Quantitative questionnaire data from patients and physicians were analyzed using IBM SPSS for Windows, versions 26 and 28. Descriptive statistical and correlation analyses (point-biserial correlations) were performed.

**Figure 1:**
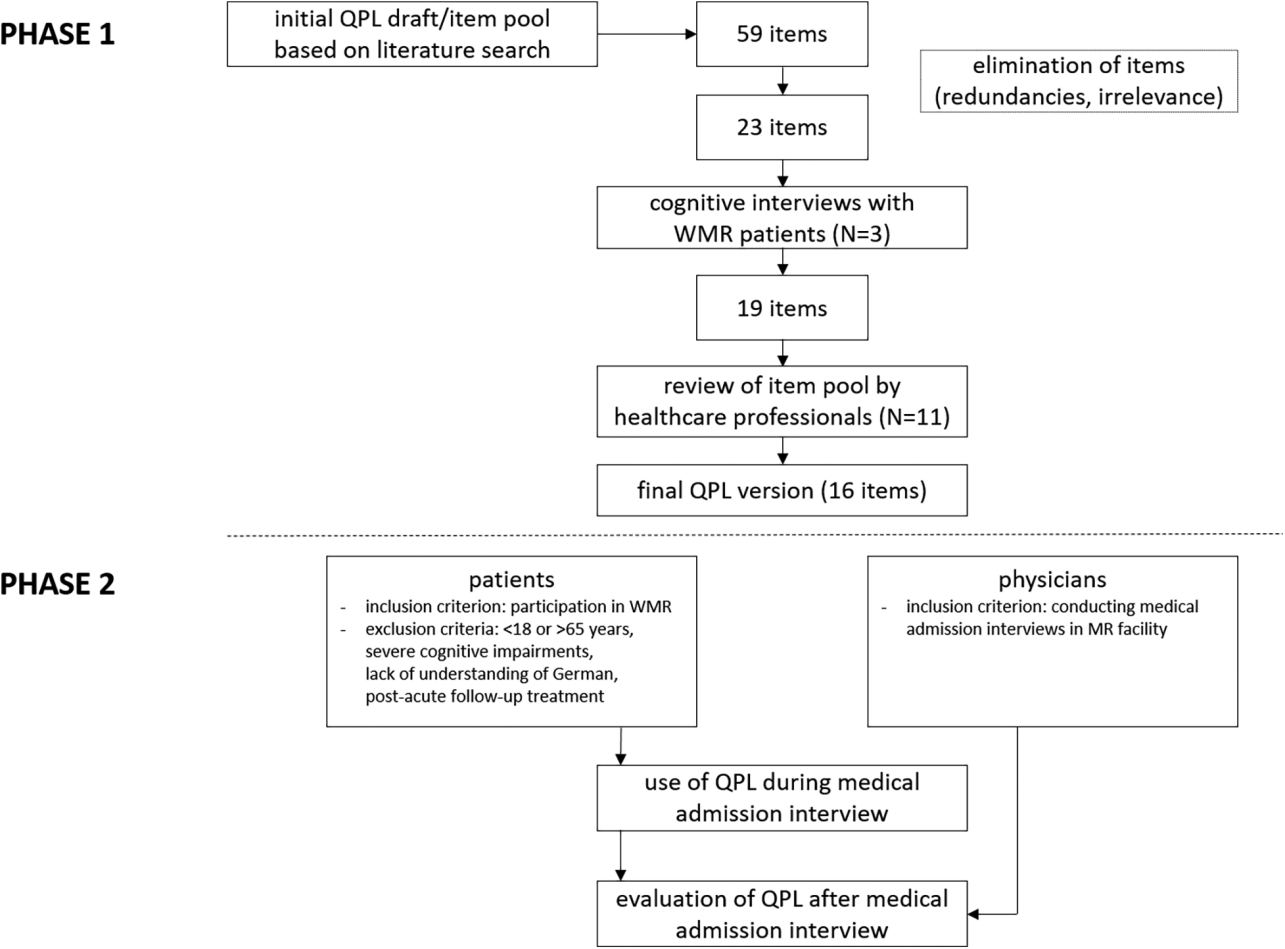
QPL development (phase 1) and evaluation (phase 2) process.

Figure 1 summarizes the process of QPL development (phase 1) and evaluation (phase 2).

## Results

### Participants

Phase 1: *N* = 3 patients could be interviewed in one MR center (one female, two male participants, mean age 53 years; duration of interviews between 30 and 90 min). Written feedback on the draft QPL was received from *N* = 11 healthcare professionals in two MR centers (professions: medicine; psychology; physiotherapy; social work; sports therapy).

Phase 2: *N* = 36 patients participated in the evaluation. Nine of them (25%) were male and twenty-seven (75%) were female. The mean age was 49.8 years (SD = 9.86; range: 31–62 years). For the majority of participants, the highest educational level was a secondary school leaving certificate or apprenticeship. Two-thirds of participants were employed full-time (*n* = 10; 28.8%) or part-time (*n* = 14; 38.9%). They were mostly employed as workers or salaried employees (Table 2).

**Table 2:** Educational and employment status of participating patients (*N* = 36).

	number (percentage)
Highest educational level	
Secondary school	29 (80.6%)
Advanced technical college certificate	3 (8.3%)
University entrance qualification	1 (2.8%)
Other school-leaving qualification	2 (5.6%)
No school-leaving qualification (yet)	1 (2.8%)
Highest vocational level	
Apprenticeship	23 (63.9%)
Vocational school	6 (16.7%)
University of applied sciences	1 (2.8%)
University	1 (2.8%)
No professional qualification (yet)	3 (8.3%)
Not stated	2 (5.6%)
Current employment status	
Full-time	10 (27.8%)
Part-time	14 (38.9%)
In vocational training	2 (5.6%)
Unemployed	10 (27.8%)
Professional position	
Worker	6 (16.7%)
Employee	27 (75.0%)
Self-employed	0 (0.0%)
Trainee/Apprentice	2 (5.6%)
Other	1 (2.8%)

Twenty-seven participants (75%) had been on sick leave in the 12 months before WMR with a mean duration of 23.6 weeks (SD = 21.56; range: 1–72 weeks). Twenty-one (58.3%) had undergone a MR program prior to the current program.

Six physicians (male: four; female: two) took part in the study with a mean age of 46.7 years (SD = 4.75). None of them had prior experience with question prompts as a tool to promote patient information.

### QPL use and evaluation by patients

Eighteen participants (50.0%) reported to have used the QPL in the medical admission interview. Four participants used all items; fourteen participants used some items. Seventeen participants (47.2%) did not use it. One person (2.8%) did not state whether the QPL was used or not.

The reasons given for not using the QPL included (questionnaire items; multiple answers possible):

• “I forgot/didn't think about the list” (*n* = 8);
• “It wasn’t clear to me what to do with it” (*n* = 7);
• “Was too cumbersome for me” (*n* = 3);
• “I already had my own notes with me” (*n* = 1)

In addition, two free-text comments were made:

• “All questions were answered by the attending physicians/therapists before I remembered the list.”
• “My questions were all answered by the doctor at the admission examination”.

The QPL was rated quite positively by those patients who used it (Table 3). The best evaluation was related to the comprehensibility of the questions on the list.

**Table 3:** Evaluation of the question prompt list by patients.

Item	*n*	M (SD)	Proportion “somewhat/fully agree” (frequencies)	Proportion “somewhat/fully disagree” (frequencies)
The QPL helped me think of everything important when talking to the doctor.	23	2.2 (.85)	19	4
The QPL helped me get all the information that was important to me.	25	2.1 (.88)	19	6
The QPL made the conversation with the doctor clear and structured.	21	2.0 (.97)	15	6
The QPL questions were easy for me to understand.	24	2.3 (.81)	21	3
The QPL questions were helpful and useful to me.	24	2.0 (1.0)	19	5
I got along well with the QPL.	23	2.1 (.85)	20	3
The purpose of the QPL was clear and understandable.	24	2.0 (1.1)	18	6

QPL, question prompt list.

Response format: 0 = fully disagree; 1 = somewhat disagree; 2 = somewhat agree; 3 = fully agree. *N* refers to the number of participants who answered/completed the respective item.

It should be noted that there were participants who evaluated the QPL despite reporting not using it (seven to nine patients, depending on the item). Presumably, these participants looked at or actually used the QPL at a different time than, for example, during the initial physician admission interview. Conversely, not all patients who used the QPL answered the evaluation items (one to four persons, depending on the item).

Study participants were also asked to indicate which of the QPL items they found helpful. Questions about obtaining “more general” information about WMR (content, importance, individual relevance) were rated as useful. Questions on co-decision-making about treatment, the importance of recovery during rehabilitation, and continuation of treatment after completion of rehabilitation were considered less helpful (see Table 5).

The information need regarding WMR before the start of rehabilitation (retrospective assessment) was moderate to low (*M* = 1.2; SD = .97; range from 0 = low need to 3 = very high need). Participants were generally satisfied with the information they received during the medical admission interview (*M* = 2.6; SD = .40; range from 0 = very dissatisfied to 3 = very satisfied).

Neither the expressed need for information (*r* = .31, *p* = .076) nor satisfaction with the information received (*r* = .06, *p* = .74) were significantly associated with the use of the QPL.

The mean score of the CARE scale of physician empathy was *M* = 3.5 (SD = .55; range from 0 = fully disagree to 4 = fully agree), indicating a high degree of perceived physician empathy during the medical admission interview. Physician empathy was significantly related to satisfaction with the information received (*r* = .39, *p* = .02), but did not correlate with the need for information (*r* = −.19, *p* = .28).

### QPL evaluation by physicians

Physician evaluations from *k* = 39 interviews were available. Thus, the number of evaluations was higher than the number of study participants (*N* = 36). In three cases, an evaluation was given for which there was no equivalent on the patient side. For pragmatic reasons, all 39 data sets were included in the analysis, since the data structure made it impossible to filter out the “surplus” three data sets or to reconstruct the exact reasons for this (it is conceivable that questionnaires were used for interviews with non-study participants or that the questionnaire was inadvertently completed twice for one interview).

In 23 cases (59%), physicians reported that the patient in question had used the QPL during the medical admission interview; in 16 cases (41%), they stated that the QPL had not been used. In four interviews, patients reported using the QPL, while in another 15 interviews, the physician asked about the use. In addition, four free-text statements were made: “[patient] had list with him/her” (mentioned twice); “[patient] had documents and letter from the pension insurance with him/her”; “(…) patient asked whether WMR makes sense for her, she had no problems at work”.

In contrast, eighteen patients stated that they had used the QPL in the medical admission interview. In this respect, there are more physician assessments (*k* = 23) based on the use of the QPL than corresponding evaluations by patients. However, as already mentioned, there were also participants who rated the QPL even though they indicated they had not used it.

In the medical admission interviews where the QPL was used by patients (to the physician's knowledge or presumption), physicians rated the list as helpful for providing information and structuring the conversation, but also perceived it to be more time-consuming (Table 4).

**Table 4:** Evaluation of the question prompt list by physicians.

The use of the QPL by the patient in the medical admission interview… (*k* = 19 interviews)	M (SD)	“somewhat/fully agree” (frequencies)	“somewhat/fully disagree” (frequencies)
…has helped to structure the conversation.	2.0 (.88)	14	5
…has helped to address/communicate all the essential information about WMR from my perspective.	2.0 (1.16)	13	6
…has helped that (in my perception) all topics that were important to the patient were addressed.	2.1 (.91)	14	5
…has contributed to the development of a good “working relationship” with the patient.	1.7 (.75)	10	9
…has resulted in a greater time commitment for me.	2.7 (.48)	19	0
…has meant that I had to change the way I conduct interviews/conversations.	1.4 (1.26)	10	9
…has resulted in certain information not being addressed that I actually wanted to discuss.	1.2 (1.4)	8	11
Using the QPL in the medical admission interview…(*k* = 4 interviews)		“somewhat/fully agree” (frequencies)	“somewhat/fully disagree” (frequencies)
… could have helped to better structure the conversation		3	0
… could have helped to address/provide even more important information about WMR		3	1
… could have helped to address more issues important to the patient.		4	0
… could have helped to establish a better “working relationship” with the patient.		3	1
…may have resulted in more effort (in terms of time, content).		0	4

QPL, question prompt list.

Response format: 0 = fully disagree; 1 = somewhat disagree; 2 = somewhat agree; 3 = fully agree.

The „skip rule“ included in the physician questionnaire for rating interviews with vs. without use of the QPL by the patient was not followed for all interviews. For four interviews with use of the QPL, only the items relating to non-use of the QPL were completed. These results are therefore shown at the bottom of Table 5.

**Table 5:** Evaluation of the question prompt list items by patients and physicians.

Item	Patients	Physicians
	Item rated as useful (number)	
1. What does WMR mean?	19	13
2. Why is WMR important to me in particular?	14	12
3. What therapies are provided in WMR (as opposed to a standard medical rehabilitation)?	22	14
4. Who can help me in WMR when it comes to sick pay, pension, and other things?	7	8
5. Can I also recover during WMR?	3	5
6. Do I receive information and feedback from diagnostic and therapeutic measures?	12	6
7. Can I also say what my problems are at work from my point of view?	8	4
8. How well does WMR fit my work situation?	10	5
9. How can WMR help me with problems with my work situation? *For example: How do I get back to work? How can I deal with stress? How can WMR help me if I have lost my job?*	10	4
10. Can I have a say in deciding which treatment/measures I receive in WMR?	4	2
11. What can I do myself to make sure that WMR helps me and is successful?	6	5
12. Are there other facilities involved in WMR besides the rehabilitation clinic?	5	3
13. Who else will be informed about my WMR program besides me? *For example: My employer, my family doctor, the company doctor*	10	4
14. What happens after WMR?	12	6
15. Should I continue treatment after WMR?	3	4
16. If I still have questions or problems after WMR: Who is responsible for me then?	8	6

The numbers given in the “physicians” column refer to the number of evaluated interviews. Multiple answers possible.

Physicians were also asked to indicate which QPL items were helpful in the interviews. While both patients and physicians found questions on general information about WMR (items 1–3) useful, there were differences with regard to feedback from diagnostics and treatment (item 6), fit of the WMR intervention to the individual work situation (items 7, 8), other institutions involved (item 13), and follow-up care/aftercare interventions (item 14). These items were rated as helpful more frequently by patients compared to physicians (Table 5).

## Discussion

The aim of this pilot study was to obtain preliminary results on the potential suitability and feasibility of a question prompt list (QPL) as a tool to support patients' knowledge and information about work-related medical rehabilitation (WMR). The QPL format was tested for the first time in the context of medical rehabilitation in Germany, a setting where there is a lack of patient-centered information and health communication tools.

Half of the participating patients reported to have used the QPL during the medical admission interview. Their evaluation of the QPL was positive: the instrument helped them to remember important things in the interview and to obtain relevant information. The QPL questions were rated as easy to understand and use. The questions on obtaining general information about WMR as an intervention were seen as particularly helpful.

Other studies show a similar proportion of about 50% of patients who actually use a QPL (5). At the same time, the majority of those who use QPLs rate them positively and as useful (5, 8, 11, 12). While these studies were conducted in oncology or other acute care settings, a similar trend could be observed for medical rehabilitation.

The main reason for not using the QPL in our study was that the list had been forgotten. The QPL had been sent to the patients together with other study documents a few weeks before the start of their rehabilitation program. It is conceivable that the instrument was often “lost” in the run-up to the rehabilitation measure, even though participants were reminded of it during participant recruitment at the beginning of their stay in the rehabilitation center and were also given the list again if needed. In the research literature, lower utilization rates for QPL were documented when they were handed out with some delay to the medical consultation (5). This is consistent with the findings of the present study. Other reasons for not using the QPL in our study were lack of clarity about its purpose and a lack of need for questions. This has also been documented in other studies (29, 30).

In our study, QPL use was not related to patient orientation in the medical admission interview (operationalized as perceived physician empathy). Previous research has found positive correlations but also no associations between QPL use and satisfaction with medical consultations (5, 7, 31–33). These inconsistent results are probably influenced by factors such as different operationalizations of patient satisfaction (or similar indicators assessing the physician-patient relationship), other influences on the physician-patient relationship, or possible ceiling effects.

Patient satisfaction with the information provided by physicians in the admission interview was high in this sample. This could indicate that rather few patients saw an (additional) need for a tool to help them obtain information. The extent to which this is the case in other, larger samples needs to be investigated in future research.

The finding that only half of the participants used the QPL in the interview with their physician could be indicative of potential “subgroups” of WMR patients who have different needs for such an instrument. However, this cannot be inferred from our data and should be examined in future studies.

Physicians rated the usefulness of the QPL in the interviews in which the instrument was used positively in terms of structuring the conversation and addressing essential information. Several evaluation studies also show that QPL tend to be rated favorably by physicians, e.g., in terms of promoting patient information and having little impact on routine procedures (9, 11, 30, 34).

At the same time, physicians stated that they had to spend more time on the interviews due to the use of the QPL. However, a longer duration of the consultation can also be beneficial, since it allows important topics to be discussed (9). Whether the increased time spent was generally perceived as unfavorable cannot be inferred from the data of our study. This could be investigated in future studies, as could a potential reduction in the number of QPL items.

### Limitations and strengths

The following limitations should be considered when interpreting the study results:

No statement can be made about the validity of the instrument as no such assessment was made in the pilot study, which focused on an initial test of its suitability from the users' perspective.

The study results are based on a small sample. The data for the QPL evaluation were collected in only one rehabilitation facility. Thus, it cannot be assumed that the data are representative of the WMR setting in general. Future studies should therefore include larger samples and be multicenter to account for a possible “clinic factor”. The latter could also include different “communication cultures” in clinics, a varying emphasis on patient-centered communication or differences in the existing communication and information provision skills of health professionals (35, 36). This may have an impact on the perceived usefulness of QPL and might be considered in follow-up studies.

As mentioned, due to pandemic-related restrictions in rehabilitation centers, a smaller number of patients than planned were included in the cognitive interviews for the further development of the QPL questions (phase 1). It is therefore questionable whether sufficient reference to the needs of WMR patients could be ensured when drafting and selecting the QPL questions. As the items themselves were created based on a literature search (an approach also used in some other QPL development studies) (10, 11), the question of what information patients want to feel well informed was only indirectly addressed in our study. It is possible that greater benefit would have been achieved if rehabilitation patients had been included in the development of the QPL items.

Another option would have been to conduct a patient survey to determine the specific information needs in WMR patients, which has been part of the development of QPL in some studies (37, 38). Although our research group had conducted a qualitative study of WMR patients' informedness prior to this study (unpublished data) and its findings had been included in the literature search, that study did not include a comprehensive needs analysis of WMR patients' information needs, which can be seen as a shortcoming.

The patient and physician questionnaires used to evaluate the QPL were developed specifically for this study, as was the case in several other studies (8, 11, 38). They are therefore not validated instruments, and their psychometric properties were not analyzed in the study. This limits their validity and informational value.

Finally, we did not assess how many and which of the QPL questions were actually asked, but rather recorded whether the list was used (in whole or in part) by patients. While such information might have contributed to a more nuanced picture of how the list was evaluated, we sought to provide the study participants with an instrument that was as short and easy to use as possible.

One strength of our study is that, to our knowledge, it represents the first application of a QPL in medical rehabilitation. This is an important setting for the treatment of chronic diseases, which represent a significant part of the disease spectrum in the population both internationally (39) and nationally (40). In the context of the German healthcare system, this pilot is important as there are gaps in the availability of simple and easily accessible information about MR for (future) patients. During development, the QPL was rated by both patients and healthcare professionals. In the subsequent evaluation, both patients and physicians in MR facilities assessed it. In this way, a multi-perspective development was undertaken.

Future research should address the validity and effectiveness of this tool. Specifically, the next steps (as planned in future studies by our research group) should include a more comprehensive formative evaluation as well as an analysis of the validity of the instrument. In this context, it will also be investigated whether there are specific subgroups of patients who have more pronounced information and support needs, e.g., depending on their health literacy level (41) or the nature or severity of their occupational problems – variables we did not investigate in this study.

A subsequent efficacy study (RCT) should investigate whether a QPL (compared to non-use or other information provision strategies) can increase the information level (or reduce information needs/deficits) in rehabilitation patients.

If the instrument proves its validity and usefulness, future follow-up studies should also address implementation issues, such as identifying (and modifying) barriers or facilitators to the use of QPL in MR or coordinating QPL with other approaches to support information delivery in routine care, e.g., by rehabilitation centers or healthcare providers.

It would be innovative to explore whether the use of QPL or similar tools corresponds to the typical communication patterns of patients and healthcare professionals in consultations or physician conversations. These patterns may be difficult to change through interventions such as QPL or may even run counter to them (42). When developing instruments to promote patient information and participation (and thus health literacy as well) in medical/work-related rehabilitation, such aspects should also be taken into account.

## Conclusion

This pilot study has provided an initial insight into the usefulness of a question prompt list as a potential tool to promote patient knowledge and information in work-related MR. The QPL was generally rated positively by both the patients using it and physicians. The relatively low number of participants who made use of the QPL is in line with other, non-rehabilitation research on QPL.

The extent to which the QPL can actually be beneficial for patients in medical rehabilitation with different levels of knowledge or health literacy cannot be deduced from the results of this pilot study and should be determined in follow-up studies.

For the specific context of medical rehabilitation in the German healthcare system, “coordination” with other approaches to support patients in obtaining information about their rehabilitation seems important. However, this may also apply to other areas of healthcare.

## Data Availability

The raw data supporting the conclusions of this article will be made available by the authors, without undue reservation.

## Footnotes

^1^A search for the combination of the search terms "question prompt rehabilitation" did not yield any (relevant) hits in the PUBMED and PSYCINFO databases at the time.

^2^At least n=40 patients per MR center should be included as study participants for the evaluation, i.e., a minimum total of n=80 to be able to statistically validate substantial correlative relationships (r>0.3). This number was considered realistically achievable by the participating rehabilitation centers during the planned survey period.
